# Synthesis and characterization of copper nanoparticle-based hydrogel and its applications in catalytic reduction and adsorption of basic blue 3

**DOI:** 10.1016/j.heliyon.2024.e25836

**Published:** 2024-02-09

**Authors:** Sultan Alam, Imran Badshah, Shahid Khan, Luqman Ali Shah, Muhammad Zahoor, Muhammad Naveed Umar, Riaz Ullah, Essam A. Ali

**Affiliations:** a Department of Chemistry, University of Malakand, Chakdara Dir Lower, 18800, Pakistan; b National Center of Excellence in Physical Chemistry (NCE), University of Peshawar, Pakistan; c Department of Biochemistry, University of Malakand, Chakdara Dir Lower, KPK, 18800, Pakistan; d Department of Chemistry, University of Liverpool, UK; e Department of Pharmacognosy, College of Pharmacy, King Saud University, Riyadh, Saudi Arabia; f Department of Pharmaceutical Chemistry, College of Pharmacy, King Saud University, Riyadh, Saudi Arabia

**Keywords:** Adsorption, Hydrogel, Copper, Kinetics, Isotherms

## Abstract

Most of the dyes used in various industries are non-biodegradable and carcinogenic in nature. Therefore, elimination of dyes from textile wastes is mandatory to safeguard the life of human, aquatic animals and aquatic plants. In this connection an effective and eco-friendly hydrogel was synthesized from acrylamide, cellulose, clay, and copper salt abbreviated as AMPS(PHE-Ce)/MC-Cu. The fabricated hydrogel was used as sorbent and catalyst for the adsorption and catalytic reduction of basic blue 3. SEM analysis showed granular texture with small holes or cracks which is basic criteria for an adsorbent surface. The results showed that the BET surface area and the Langmuir surface area were, respectively, 27.87 and 40.32 m^2^/g. The FTIR analysis confirmed the synthesis of hydrogel, as is evident from peaks at 3500, 3439, 2996, 2414, and 1650 cm^−1^, which indicated the presence of OH or NH, -C-O-C-, CH_3,_ (C=O), C–N bonds correspondingly. Thermal stability was confirmed by TGA analysis where weight loss in three stages has been observed. The presence of copper was confirmed through EDX (5.02%) indicating the incorporation of cupper nanoparticles in hydrogel surface. The high adsorption capability of 1590 mg/g as recorded for basic blue-3 dye indicates it to be an efficient adsorbent. The swelling behavior characterized by Fickian diffusion up to 7898% clearly indicated significant swelling. Pseudo 2nd-order kinetics and the Langmuir isotherm models were more fit in unfolding kinetics and isothermal data indicating chemisorption with monolayer sorption as evident from the high R^2^ values (0.999) of each model. Thermodynamics considerations indicated that the adsorption process is endothermic with a positive enthalpy value of 1371.32 Jmol^-1^. The positive entropy value of 19.70 J/mol.K signifies a higher degree of disorder at the solid-liquid interface. The findings provided a valuable insights into the hydrogel's capacity to adsorb cationic dyes and reduce them catalytically, pointing towards its potential applications in addressing environmental challenges.

## 1 Introduction

A number of commercial dyes are highly persistent in ecosystem and resists natural break down processes under earth climatic conditions, emphasizing the need to address the effects of dye consumption and manufacture on the environment [1]. The main contributor to water pollution are the industrial discharges from various sectors, including metals, plastics, food, pharmaceuticals, and dyes [2,3]. The industrial wastewaters often carry harmful pollutants among which dyes are the serious risk to aquatic and terrestrial organisms. Unlike pigments that remain insoluble, dyes have the ability to dissolve therefore, interfering the photosynthesis aquatic plants which then are unable to provide necessary oxygen for aquatic organisms [3,4]. That is why textile industry is considered to be a major contributor to environmental pollution as compared to other sector's addition in pollution shares of aquatic environment [5,6]. Dyes find their extensive uses in many sectors such as tanneries [7,8], paper and pulp manufacturing [[9], [10], [11]], food processing, electroplating [12,13], dyeing [14,15], and may more, releasing large volumes of colored wastewater into the environment. The initial signs of the dyes pollution in water is foul-smelling [16] as encounter in densely populated areas. Annually, approximately two million tons of dyes are synthesized globally, finding use in various industries in coloring plastics, food item [17,18], paper, textiles fibers [19,20], cosmetics [21], inks, and pharmaceuticals [22] as mentioned above. Addressing the challenges in handling water pollution caused by dyes is a tedious job. Also, implementation of effective preventive measures is a time-consuming process. Unfortunately, a substantial portion of the dyes in useful works is wasted and it has been found that approximately 15%, ends up in the environment. Dyes not only decrease dissolved oxygen levels and block sunlight from reaching the water but also, can introduce harmful chemicals into the human food chain [[17], [18], [19], [20], [21], [22]].

For the effective reclamation of substances like basic blue-3 (BB-3) and other organic pollutants from wastewater, various methods are in use. Some of these methods involve techniques like adsorption and desorption [23], radiation, ozonation [18,[19], [24], [25]], photocatalysis [26], ion exchange [27], membrane separation [28], osmosis [29], electrochemical processes [30], and even the use of biochemical material as adsorbents. However, many of these existing approaches can be expensive and may not completely eliminate all harmful substances from water loaded with dyes. As an effort to make these troublesome dyes less harmful, a straightforward and environmentally friendly approach has been developed, which combines adsorption with a chemical process. However, this process can be somewhat slow when used on its own. To overcome this challenge, a creative solution has been devised by incorporating tiny metal (Cu) particles into a special polymer network. The process is significantly accelerated by the presence of metal particles acting as catalysts, and everything is kept well-organized and under control by the polymer network. When combined, this innovative approach results in an enhancement of the speed and efficiency of the reduction and adsorption process [31].

In this study, a distinctive material was created by blending two substances, polyhydroxyethyl cellulose and acrylamide, recognized for their pH-responsive properties. Additionally, small particles of Mosiv type 5-A clay were introduced, and copper nanoparticles were integrated into this mixture. The primary objective was to examine how the quantity of copper nanoparticles affects particle size, pH sensitivity, and their efficiency in degrading and adsorbing Basic Blue-3 (BB-3) dye. Furthermore, research was conducted to determine if these components could be reused in the chemical process. Hydrogels, particularly when modified with substances such as acrylamide, cellulose, clay, and copper nanoparticles, are preferred for various applications. They are favored due to their high water content, biocompatibility, tunable properties, and versatility, enabling adjustments to be made. Responsive to environmental changes like temperature or pH, they are utilized for environmental cleanup, effectively absorbing pollutants from water. An intriguing aspect is their potential for frequent regeneration and reuse, making them a sustainable option for absorption purposes [32].

The project's objectives include the enhancement of understanding hydrogels containing copper nanoparticles, with emphasis placed on the creation and study of these hydrogels. The primary objective of the study was to determine the reduction and removal capacity of synthesized hydrogel for Basic Blue 3 dye from wastewater. Key objectives involve the development of an efficient synthesis method, characterization of the hydrogels, and exploration of their practical applications.

## 2 Experimental

### 2.1 Equipment and reagents utilized

In the present work, a variety of chemicals were employed, including 2-acrylamido-2-methylpropanesulfonic acid (AMPS), mosiv type 5-A clay (MC), polyhydroxyethyl cellulose (PHE-Ce), copper salt (CuCl_2_.2H_2_O), N, N′-Methylenebisacrylamide (MBA), ammonium persulfate (APS), sulfuric acid (H_2_SO_4_), KOH (potassium hydroxide), (HCl) hydrochloric acid, (NaOH)sodium hydroxide, and Basic Blue-3 dye (BB-3). These chemicals were of high quality to ensure precise and consistent outcomes in our experiments. Double-distilled water was used for the studies in order to maintain controlled conditions.

### 2.2 Fabrication of AMPS(PHE-Ce)/MC-Cu hybrid hydrogel

A specialized gel was developed through the combination of AMPS (2-acrylamido-2-methylepropanesulfonic acid), polyhydroxy ethyl cellulose (PHE-Ce), and mosiv type 5-A clay (MC) in a specific ratio (69% AMPS, 26% PHE-Ce, and 5% MC) using the free radical polymerization process [33]. To render the mosiv clay (MC) suitable for the intended purposes, it was cleaned using a combination of double-distilled water and peroxide. This cleaning process aimed to eliminate any impurities that could be either soluble or of organic origin. The purified MC was then crushed into fine particles (145–185 mesh) and dried for subsequent use. 0.552 g of MC were combined with 70 mL of distilled water, and the mixture was continuously agitated at 500 rpm overnight to achieve a homogeneous dispersion of mosiv clay particles in water. Following this, the monomer and mosiv clay solutions were mixed, and ammonium persulfate, also known as APS, was added to the mixture. The entire blend was then placed in an oven at 60 ± 1 °C to undergo the required processes [19]. The hybrid hydrogel was quickly formed, and the reaction was sustained for 2.5 h to guarantee the full utilization of any remaining monomers (unreacted). The subsequent product underwent purification through overnight immersion in MQ water. Subsequently, a vacuum oven was used to dry the hybrid hydrogel at 42 °C and 0.76 kPa of pressure and stored for its application in the sorptive removal of Basic Blue-3.

To introduce copper nanoparticles, a certain amount of copper salt (CuCl_2_.2H_2_O) was dissolved in water to create a solution with a (0.003 M) concentration of the salt. A portion of this solution was taken, and the prepared hydrogel was added, followed by stirring for 20 h at a controlled temperature of 27 ± 20 °C. Subsequently, a sodium borohydride (NaBH_4_) solution was prepared, and copper nanoparticles were introduced for 5 h. As a result of this process, the hydrogel's white color turned brown, signifying the presence of copper nanoparticles with reduction qualities. The hydrogel was coded as AMPS(PHE-Ce)/MC-Cu.

### 2.3 Sorption experiment

The interaction between the AMPS(PHE-Ce)/MC-Cu hybrid hydrogel and BB-3 dye was investigated using the batch adsorption method. The experiment involved placing 0.0503 g of the hydrogel in a reagent bottle (RB) with 15 mL of a specific BB-3 dye concentration. Sulfuric acid (H_2_SO_4_) or Sodium hydroxide (NaOH) was used to alter the mixture's pH. The RB was then positioned in a water bath and rotated at 217 revolutions per minute (rpm) for a set time duration. Subsequently, the solution underwent filtration, and the concentration of BB-3 was determined using a UV–visible spectrophotometer. To ensure precision, this procedure was repeated thrice, and the outcomes were derived from the mean values obtained.

#### 2.3.1 Sorption kinetics

The kinetic study was conducted at three distinct temperatures (283, 293, and 303 K) with varying durations ranging from 5 to 50 min [19]. The aim was to investigate the absorption of B Blue-3 by the AMPS(PHE-Ce)/MC-Cu hybrid hydrogel. The study proceeded as follows: Six reagent bottles (RBs) were employed, each containing 15 mL of a BB-3 (0.001 M) solution and 0.0503 g of the superabsorbent hydrogel. The RBs containing dye solution and hydrogel were positioned within an automated water bath and subjected to agitation at a velocity of 217 rpm. Samples were extracted at intervals of 5, 10, 15, 20, 25, 30, 35, 40, 45, and 50 min for analysis.

#### 2.3.2 Sorption isotherm

To initiate the experiment, 0.0503 g of the sorbent were accurately measured and amalgamated in dyes in each reagent bottle containing 15 mL BB-3 solutions with various starting concentrations between 0.00053 and 0.0047 M which were individually prepared and put into each reagent bottle. Following the initial treatment, the pH of the solutions within each RB was adjusted to a neutral level of 7. The RBs were then immersed in a temperature-controlled water bath set to 303 K (approximately 30 °C), where they underwent agitation at a velocity of 217 rpm for a predetermined duration, as established through kinetics experiments.

### 2.4 Best-fitting isotherm model estimation Chi square test

The Chi-square test, symbolized as χ^2^, is like a tool in statistics that helps us figure out how closely our real-world observations match what we'd anticipate from a model Equation as present in Eq. (1) below. To put it simply, it adds up the squared differences between what we measured in an experiment and what we'd predict according to a theoretical model. This test gives us a sense of whether there's a big difference between what we see and what we'd expect.

(1) $$\chi^{2}₌\sum \frac{\left(q_{e,exp}-q_{e,cal}\right)2}{q_{e,cal}}$$

### 2.5 The effect of pH on the sorption process

A specific volume of dye solution with a constant concentration, as mentioned earlier, was mixed with specified amount of adsorbent. The solutions were maintained at various pH levels, ranging from 2.2 to 10.4. The subsequent experimental procedure followed the same steps as previously described.

### 2.6 Thermodynamics study

To examine the effect of temperature on the sorption process under investigation, the sorbent (0.0503 g) was exposed to 15 mL of dye solutions at three different temperatures: 283 K, 293 K, and 303 K with uniform stirring. The experimental procedure remained consistent with the earlier description. The collected data were used to determine the values of changes in Gibbs free energy (ΔG°), enthalpy (ΔH°), and entropy (ΔS°).

### 2.7 Catalytic activity of AMPS(PHE-Ce)/MC-Cu

An experiment was conducted to measure the potential catalytic activity of the AMPS(PHE-Ce)/MC-Cu composite system while reducing basic blue 3 dye. This reduction was achieved using NaBH_4_ in a water-based solution. The experiment was carried out as follows: A solution containing 0.01 mM (mM) basic blue 3 was combined with another solution containing 32 mM NaBH_4_, resulting in a 2 mL total volume. The mixture was introduced into a cell of quartz with a path length of one cm. Subsequently, 0.4 g of the AMPS(PHE-Ce)/MC-Cu hydrogel catalyst was added to the cell. The progress of the reaction was monitored using a UV/visible spectrophotometer.

### 2.8 Swelling effect study

As mentioned before the study aimed to examine the response of a specialized hydrogel to varying levels of acidity or pH, ranging from 4.6 to 10.2. These experiments were conducted at room temperature, approximately 25 ± 2 °C, and were carried out as follows: A small section of the hybrid hydrogel, measuring just 0.53 square centimeters, was initially carefully dried in an electric oven set at 82.7 °C to remove all moisture. Subsequently, the mass of this dry section was precisely measured using a digital scale. The dry section was then immersed in water and allowed to soak for a period of 25 h. After this soaking duration, the section's weight was re-measured to determine the extent of weight gain resulting from water absorption. To assess how the hydrogel expanded or swelled under different pH conditions, a specific Equation (Eq. 2) was employed [34].

(2) $$\mathrm{Percent}\;\mathrm{Swelling}=\frac{M_{\mathrm{sw}}-M_{\mathrm{dr}}}{M_{\mathrm{dr}}}\times 100$$

Where $M_{\mathrm{dr}}$ stands for the weight of the dry pieces of the hydrogel we created, and $M_{\mathrm{sw}}$ represent the weight of those same pieces after they have absorbed water and swelled.

### 2.9 The characterization of hydrogel

Several analytical methods were employed to assess the properties of the prepared hydrogel. FTIR measurements were conducted to identify specific functional groups present in the hydrogel's structure. Subsequently, SEM and BET analyses were done to scrutinize the surface characteristics and porosity of the hydrogel. To evaluate its thermal stability, TG/DTA analysis was carried out using a TGA-50 Shimadzu instrument. Elemental analysis of the prepared hydrogel was conducted using Energy-Dispersive X-ray Spectroscopy (EDX) with the Oxford energy-dispersive X-ray spectrometer. To determine dye concentration, a UV–visible spectrophotometer (1800 Shimadzu), Scientific Instruments Inc., headquartered in Kyoto, Japan) was employed to measure light absorption at a specific wavelength (654 cm^−1^). Consistent temperature conditions for adsorption study were maintained using a water bath shaker (thermostatic) to ensure the reliability and accuracy of results.

### 2.10 Reusability

The adsorbent that had been in contact with the dye solution was regenarated with the help of solvent extraction technique. In this process, the composite material was treated with 10.6 mL of acetone to remove the adsorbed dye from the hydrogel. The hydrogel was successfully reused as an adsorbent for six consecutive cycles.

## 3 Result and discussion

### 3.1 Reaction pathway for synthesis of AMPS (PHE-Ce)/MC-Cu hydrogel

The development of the AMPS (PHE-Ce)/MC-Cu sorbent involves several critical processes. Initially, ammonium persulfate (APS) is utilized, breaking down into radicals recognized as sulfate anion (SAR). These SARs interact with the PHE-Ce backbone, facilitating the removal of hydrogen atoms and the formation of an alkoxy (RO) macro radical initiator. Subsequently, the RO radicals are targeted by the double bonds in AMPS, leading to the creation of new reaction sites in the process [34]. The double bonds in the monomers are targeted by these sites, leading to the generation of more active sites. The successive reaction keeps going until a lengthy polymeric chain is created. At the same time, N, N-methylene bisacrylamide (MBA) forms a three-dimensional polymeric structure by acting as a connector (cross linker) between the expanding polymeric chains. In the final stage, the active sites attract the polar components of the gel's MC (mosiv clay) and copper nanoparticles [19]. This attraction enhances the structural stability of the hydrogel, culminating in the desired graft copolymeric material, as illustrated in Scheme S1.

### 3.2 Sorption sensitivity to pH variations

**Fig. 1:**
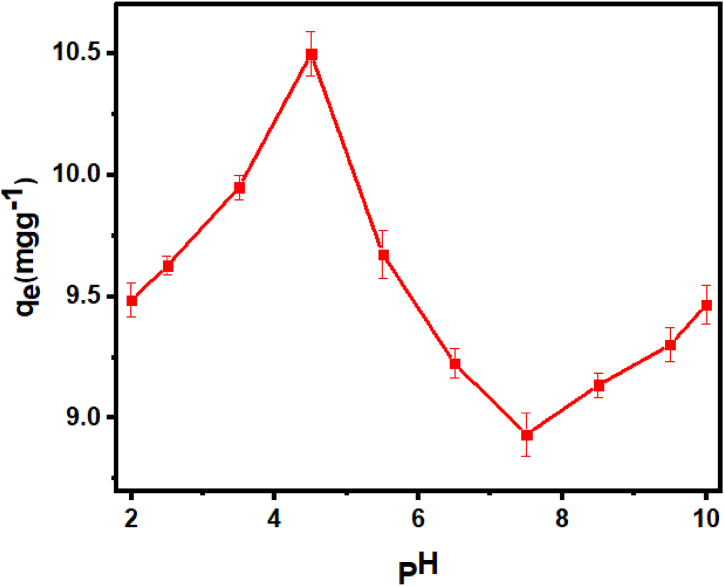
Plot q_e_ verses pH of media containing AMPS(PHE-Ce)/MC-Cu.

It is evident from Fig. 1 that the hybrid sorbent's capacity to eliminate BB-3 (q_e_) varies in response to variations in the medium's pH. The sorption of BB-3 is quite modest at pH 2.2, but it increases quickly until pH 5.5. Following this, the drop resumes until pH 8.1 before abruptly increasing from pH 8.3 to pH 10.4. The asymmetric pattern observed is attributed to structural alterations in BB-3 and the hydrogel under acidic and alkaline conditions. At low pH values, the surface of the AMPS(PHE-Ce)/MC-Cu hydrogel becomes positively charged. This leads to columbic repulsion between the cationic BB-3 dye molecules and the positively charged hydrogel, thus limiting the binding capacity of the basic dye. Conversely, as the pH of the solution increases, the hydrogel's outer surface shifts towards a negative charge, creating an attraction between the basic dye molecules and the adsorbent surface. However, at very high pH values, the hydrogel surface of BB-3 may become negatively charged, causing the dye molecules to be repelled by it. Therefore, the optimum pH for the adsorption of BB-3 onto the AMPS(PHE-Ce)/MC-Cu hydrogel was considered as pH 4.5.

In summary, pH has a crucial role in the sorption process. In an alkaline environment, the AMPS(PHE-Ce)/MC-Cu hydrogel deprotonates, making surface neutral (pKa = 5.9) as shown in Scheme S2a. In a nutshell adsorption is influenced by the equilibrium between BB-3 protonation and COOH deprotonation in the hydrogel. The existence of both deprotonated adsorbent and protonated BB-3 in significant amount facilitating electrostatic interaction, resulting in the maximum sorption potential at pH 5. Conversely, when the pH is lower than the pKa of BB-3 (pKa = 4.5) it become more positively charged, enhancing its ability to bind with the anionic hydrogel (Scheme S2b) [34].

### 3.3 Adsorption kinetics study of Basic Blue-3

**Fig. 2:**
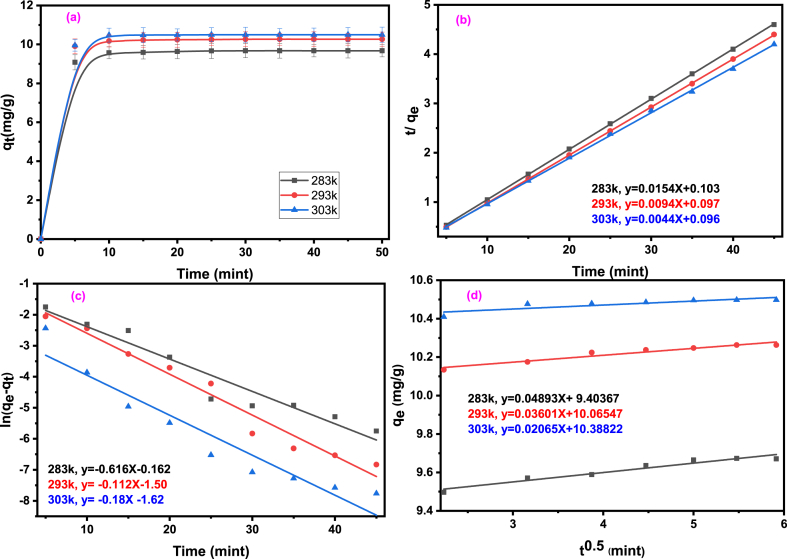
Kinetic study (a) effect of contact time (b) Pseudo- 2nd order model (c) pseudo-first order kinetic (d) and Intra particles diffusion model for adsorption of BB-3 dye on AMPS(PHE-Ce)/MC-Cu hydrogel.

To evaluate the sorbent's performance and understand how sorption process proceeds, it is important to study its kinetics. The results of kinetics study are shown in Fig. 2a. Experiments conducted at various temperatures reveals that initially, the sorption rate is fast because there are numerous available sites for adsorption. However, after about 5 min, the sorption rate significantly decreases. This slowing down happens because the dye diffuses more slowly now into the superabsorbent, and there are fewer available sites for adsorption. This factor is crucial to reach to equilibrium of the process [35,36]. Moreover, optimum temperature for adsorption was found to be 303 K.

To examine how quickly BB-3 is absorbed, the obtained data was fed to equations of pseudo-first order (Equation (3)), pseudo-second order (Equation (4)), and the intra-particle diffusion models (Equation (5)).

(3) $$\ln\left(q_{e}-q_{t}\right)=\ln\;q_{e}-k_{1}t$$

(4) $$\frac{t}{q_{t}}=\frac{1}{q_{e}}t+\frac{1}{k_{2}q_{e}^{2}}$$

(5) $$q_{t}=k_{\mathrm{diff}}t^{0.5}+c$$

These equations are crucial in determining how much amount of BB-3 is absorbed at specific times (q_t_) and when the sorption process touches to equilibrium (q_e_). It also allows us to calculate the rate constants for the pseudo-first order (k_1_) and pseudo-second order (k_2_) reactions.

Table 1 displays the estimated values of k_2_ and q_e_, obtained at three distinct temperatures. These approximated values were determined by examining the slope and intercept of the t/q_t_ vs t plot, as depicted in Fig. 2b, which was generated using a pseudo-2nd order equation. The results of the experiment are presented in Table 2 and Fig. 2c, which also depict the slope and intercept of the ln(q_e_–q_t_) vs. t plots at different temperatures. These values were utilized to calculate the kinetics parameters of q_e_ and k_1_. Furthermore, the constants K_diff_ and C were estimated from Equation (5) by analyzing the slope along with the intercept of graphs q_t_ versus t^1/2^. These values are essential for gaining insights into how the sorption process occurs, particularly in the context of treating contaminated water, as shown in Table 1 (Fig. 2d).

**Table 1:** Calculated kinetic parameters for the sorption of BB-3 on AMPS(PHE-Ce)/MC-Cu gel using various models.

Temperature (K)	Kinetic parameters of pseudo first order (k_1_)				
	K_1_ (mint)^−1^	q_e_ (mg g^−1^) expe	q_e_ (mg g^−1^) cal	χ2 test	R^2^
283	0.163	9.715	0.5345	0.0093	0.823
293	0.112	10.29	0.233	0.0091	0.984
303	0.176	10.51	0.199	0.0088	0.847
Temperature(K)	**Parameters of pseudo second order (k**_**2**_**)**				
	K_2_ (mg g^−1^ mint)^−1^	q_e_ (mg g^−1^) expe	q_e_ (mg g^−1^) cal	χ2 test	R^2^
283	6.789	9.715	9.673	0.0071	0.999
293	10.4	10.29	10.262	0.0071	0.999
303	21.7	10.51	10.497	0.0069	0.999

Temperature(K)	Parameters of intra particles diffusion			
	*K*_*diff*_	C	χ2 test	R^2^
283	9.403	0.935	0.0097	0.936
293	10.065	0.924	0.0081	0.925
303	10.388	0.702	0.0099	0.702

**Table 2:** Sorption Isotherm Parameters for BB-3 onto AMPS(PHE-Ce)/MC-Cu gel.

Langmuir isothermal parameter					
Kelvin Temperature	q_m_ (mgg^−1^) Expe	q_m_(mgg^−1^) Calc	$K_{L}$ (Lg^−1^)	χ2 test	R^2^
283	932.23	1490	3.51	0.0067	0.997
293	1016.24	1660	3.23	0.0066	0.999
303	1043.60	1590	3.78	0.0066	0.999
Freundlich isothermal parameter					
Kelvin Temperature	**K**_**F**_**(mg g**^**−**^**^1^)**	$\frac{1}{n}$**(L g**^**−**^**^1^)**	n	**χ2 test**	**R**^**2**^
283	20.53	0.626	1.60	0.0074	0.973
293	18.44	0.658	1.52	0.0072	0.967
303	23.34	0.623	1.61	0.0072	0.965

Temkin isothermal parameter				
Kelvin Temperature	**b (jmol**^**−**^**^1^K**^**−**^**^1^)**	$K_{T}$**(**${mg\;g}^{-1\;})$	**χ2 test**	**R**^**2**^
283	7.21	34.32	0.0074	0.954
293	6.84	33.19	0.0073	0.961
303	7.03	34.85	0.0074	0.956

In a net shell it was found that the pseudo-2nd-order kinetic model provides a much well fit for our data compared to the pseudo-1st-order model. This is evident from the significantly higher R^2^ values and lower chi square value estimated from pseudo-2nd-order plots. Furthermore, the theoretically q_e_ calculated values of pseudo-2nd-order equation closely match the lab experimental q_e_ values, representing the high efficacy of our sorption process for BB-3. However, there is a noticeable discrepancy between the experimental and theoretically calculated values of q_e_ calculated from the pseudo-1st-order equation, suggesting that this model is not a good fit for our data. It is crucial to know how sorption works and to identify the rate-limiting step from such types of data under optimal conditions [37,38].

### 3.4 Adsorption isotherm study of Basic Blue-3

**Fig. 3:**
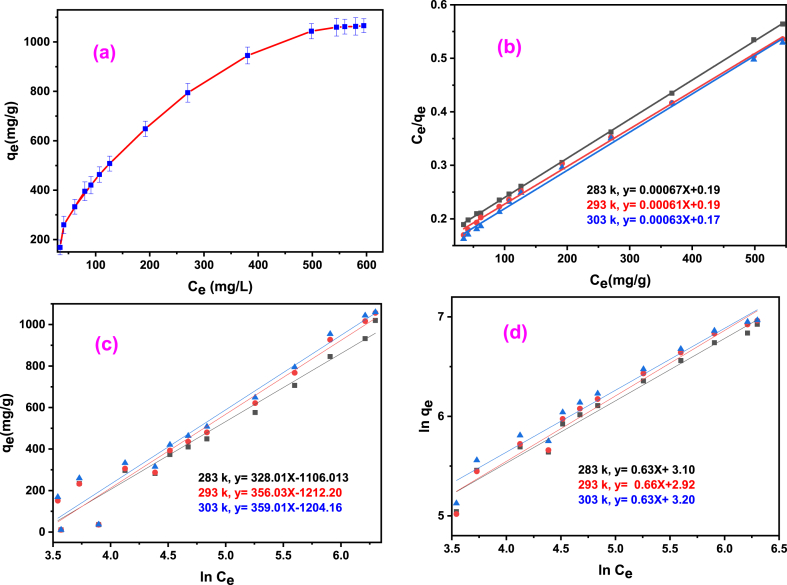
The adsorption performance of BB-3 on the AMPS(PHE-Ce)/MC-Cu hydrogel; Giles isotherm (a) Langmuir plot (b) Temkin plot (c) Freundlich plot (d).

When designing a wastewater treatment system, it is crucial to know about the sorption isotherm, which helps in determining the maximum sorption capacity. The results isotherm studies depicted in Fig. 3a, showed a distinctive relationship between the remaining BB-3 at equilibrium (q_e_) and its residual concentration (C_e_). Initially, the curve exhibits a steep increase, then there is a gradual decline until reaching a saturation point, indicating that all available sorption sites are filled with BB-3 molecules, and there was no further increase in sorption.

Importantly, the adsorbent used in this study demonstrated a remarkable sorption capability, reaching to q_max_ = 1590 mg g^−1^ as indicated in Table 2 from the higher R^2^ value and lower chi square test values indicated the best fitting of Langmuir model with respect to other models tested. L-type of isotherm model is shown in Equation (6).

(6) $$\frac{C_{e}}{q_{e}}=\frac{1}{K_{l}q_{\max}}+\frac{C_{e}}{q_{\max}}$$

Table 2 contains data regarding the maximum sorption potential (q_max_) in mgg^−1^ and the K_l_ is the Langmuir constant in Lg^−1^, their values were obtained from the linear plot of $\frac{C_{e}}{q_{e}}$ Vs. $C_{e}$, as shown in Fig. 3b. Furthermore, separation factor (R_L_ the) was calculated using K_l_ value, utilizing Equation (7). This factor is critical in determining whether the sorption process is favorable, unfavorable, or irreversible.

(7) $$R_{L}=\frac{1}{1+k_{l}C_{o}}$$

The R_L_ values, ranging from 0.375 to 0.136 and value < 1, clearly indicates that the adsorption of BB-3 by the hybrid hydrogel was favorable [39,40].

Temkin isotherm model was also apply to data shown in Equation (8).

(8) $$q_{e}=\frac{RT}{b}\ln\;K_{T}+\frac{RT}{b}\ln\;C_{e}$$

The Temkin equation utilizes R (the gas constant), T (the temperature in Kelvin (K)), and qe (the amount of sorbed BB-3). The Temkin isotherm model considers the interactions between the adsorbent and adsorbate. This model involves a linear decrease in the sorption heat for each molecule in the layer with coverage, rather than a logarithmic decrease. This is achieved by eliminating low and high concentration values [41].

After examining the Temkin plot presented in Fig. 3c, the Temkin constant (K_T_) and sorption enthalpy (b) were determined by analyzing the intercept and slope. These values are enumerated in Table 2.

The data was also fed into the Freundlich isotherm model, as described by Equation (9).

(9) $$\ln\;q_{e}=\ln\;k_{F}+\ln\frac{C_{e}}{n}$$

In the equation, $q_{e}$ (mgg^−1^) represents the equilibrium concentration of sorbed BB-3, while $C_{e}$ (molL^−1^) is the remaining concentration of BB-3 in the solution. In order to calculate the Freundlich parameters and adsorption capacity, including K_F_ (mgg^−1^) and n, a plot of lnq_e_ against ln $C_{e}$ as shown in Fig. 3d was constructed. The parameters (calculated) are sum-up in Table 2. The effective and cooperative nature of BB-3 sorption onto the hybrid hydrogel is reflected in the value of n.

Upon evaluating the data with the Langmuir, Temkin and, Freundlich models, it was evident that the Langmuir model offered a better fit, as evident by the higher R^2^ value. This implies that BB-3 primarily binds to the hydrogel composite through electrostatic bonds formed between negatively electric charged sites (COO-) on the hybrid hydrogels and the positive BB-3 dye molecules.

### 3.5 Thermodynamic property for adsorption of BB-3 AMPS(PHE-Ce)/MC-Cu on hydrogel

Examining the thermodynamics is a fundamental aspect of understanding how adsorption works. The effect of temperature on the elimination of BB-3 by AMPS(PHE-Ce)/MC-Cu adsorbent was studied from 283 K to 303 K, with all other variables held constant. Thermodynamic parameters, which include alterations in Gibbs energy (ΔG), ΔS the entropy and enthalpy (H) related with the adsorption of selected dye were estimated by means of Equations (10), (11)).

(10) $$\Delta G{}^{\circ}=-RTlnK_{d}$$

Where $k_{d}=\frac{q_{e}}{C_{e}}$.

(11) $$\ln\;\left(K_{d\;}\right)=\frac{{△S}^{0}}{R}-\frac{{△H}^{0}}{\mathrm{RT}}$$

**Fig. 4:**
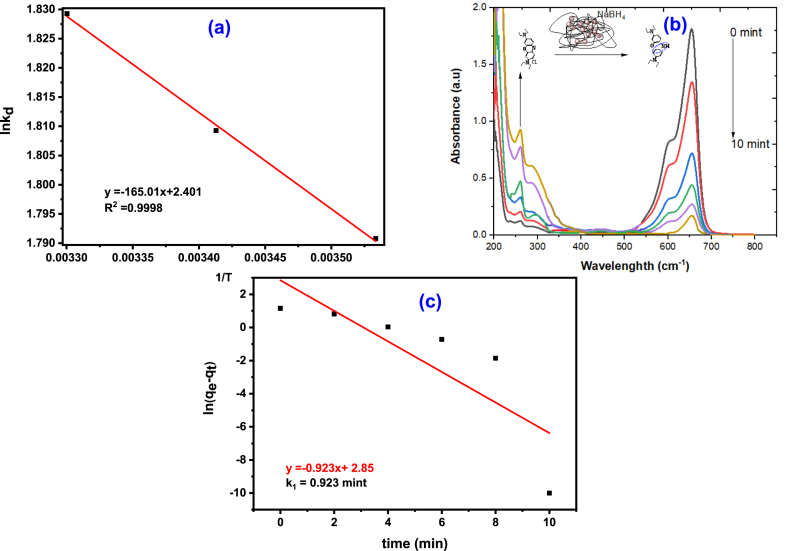
(a) Van't Hoff plot (b) UV–visible spectra for reduction of BB-3 by NaBH_4_ (c) Pseudo 1st order kinetic model for AMPS(PHE-Ce)/MC-Cu hydrogel.

In Fig. 4a, shows ln(K_d_) (the natural logarithm of the equilibrium constant) against 1/T (the reciprocal of temperature) plot [42]. The estimated thermodynamic parameters are summarized in Table 3. The Gibbs ΔG° values, are negative at many temperatures (283, 293, and 303 K) −4213.45, −4407.34, and −4608.22 J/Mol. The negative sign indicates that the adsorption process of BB-3 onto the AMPS(PHE-Ce)/MC-Cu adsorbent is energetically favorable and spontaneous. The positive value of ΔH° (1371.32 J mol⁻^1^) suggests the adsorption process to be an endothermic one. The positive value of ΔS° (19.70 J mol⁻^1^ K⁻^1^) indicates a strong attraction between the BB-3 molecules and the AMPS(PHE-Ce)/MC-Cu hydrogel adsorbent surface. It also suggests that there's an rise in disorder at the solid solution interface throughout the adsorption process [43,44].

**Table 3:** Thermodynamics parameters for BB-3 on AMPS (PHE-Ce)/MC-Cu hydrogel.

ΔG°(J/mol)			ΔH° (J/mol)	ΔS°(J/mol.K)
**283K**	**293K**	**303K**		
−4213.45	−4407.34	−4608.22	1371.32	19.70

### 3.6 Catalytic activity

**Scheme 1:**
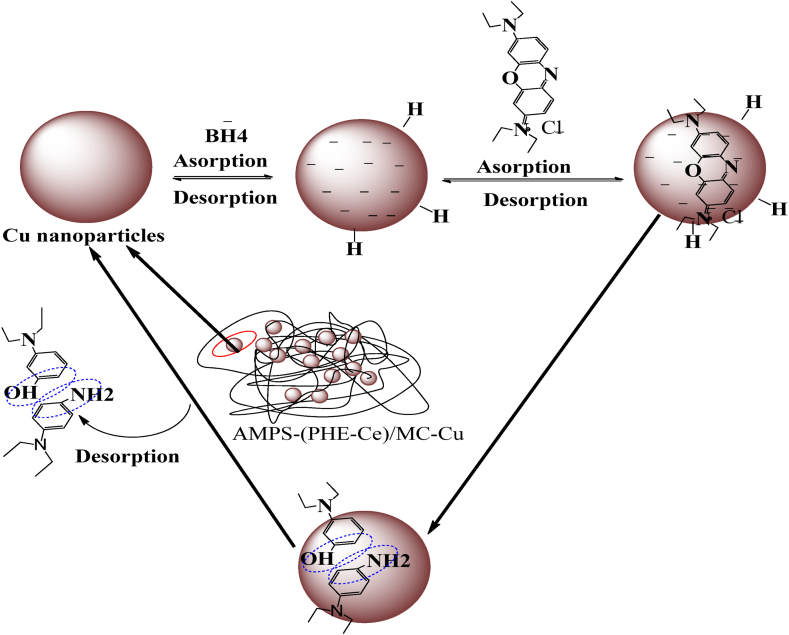
Langmuir- Hinshelwood mechanism for reduction of BB-3.

The AMPS(PHE-Ce)/MC-Cu material was tested as a catalyst for reducing BB-3 using NaBH_4_ (sodium borohydride), a water-based solution at 25 °C (Room emperature). The process of reduction occurs on the copper nanoparticles surface and follows the Langmuir Hinshelwood model. This is a basic justification, that how this catalytic activity operates, as shown in Scheme 1 [45]. Borohydride ions first attach to the surface of copper (Cu) nanoparticles during the catalytic process. leading to the release of hydrogen gas. Then, the molecules of BB-3 molecules also attached to the copper nanoparticle. Notably, both of these processes are reversible, and allowing BB-3 molecules to easily adsorb and desorbed from the copper nanoparticle surface.

The key step in this sequence is the reduction reaction taking place on the surface of copper nanoparticles. During the process of reduction the dye molecules becomes colorless. After the reduction reaction is completed, the colorless BB-3 dye molecules separate from the surface of the copper nanoparticles, effectively cleansing the surface and making it available for subsequent reactions. A unique sorption peak at 654 cm^−1^ is exhibited by BB-3, which is attributed to the presence of a chromophore within its crystal structure. Initially, even after a significant amount of time has elapsed, no discernible change in the color or absorption amount of BB-3 is observed in the presence of simply NaBH_4_ (sodium borohydride). However, upon introducing a small amount (0.4 g) of the synthesized AMPS(PHE-Ce)/MC-Cu hydrogels into the reaction mixture, a remarkable transformation takes place. All of the dark blue colors in the reaction mixture disappear, indicating the successful reduction of the BB-3 dye. The removal of these colors demonstrates the decrease in BB-3 dye concentration. UV–visible spectroscopy is employed to meticulously monitor the progression of the reaction. Changes in the BB-3 absorption spectra can be tracked by scientists using this analytical method. It becomes evident that the AMPS(PHE-Ce)/MC-Cu material facilitates the reduction of BB-3, as the colorless dye molecules detach from the surface of the copper nanoparticles, affirming the occurrence of the reduction process.

The primary absorption peak at 654 cm-1 progressively disappears, as seen in Fig. 4b–and a new peak about 260.5 cm^−1^ emerges in the BB-3 spectrum. This appearance of a new peak indicates that the azo group within the Basic Blue-3 (BB-3) molecules has been reduced.

Plotting the natural logarithm of the disparity in the amount adsorbed at time t $q_{t}$ and the amount adsorbed at equilibrium $q_{e}$ over time (Fig. 4c) reveals a linear connection. The reaction appears to follow pseudo-first-order kinetics with respect to BB-3, based on this linear pattern. Given the comparatively large percentage of sodium borohydride (NaBH_4_), this is in line with expectations. The rate constant (k_1_) for the reaction can be found by analyzing the slope of this linear curve, and it turns out to be 0.922 min⁻^1^. It simply indicates that pseudo-first-order kinetics govern the decrease of BB-3.

### 3.7 Characterization

#### 3.7.1 Swelling experiment

**Fig. 5:**
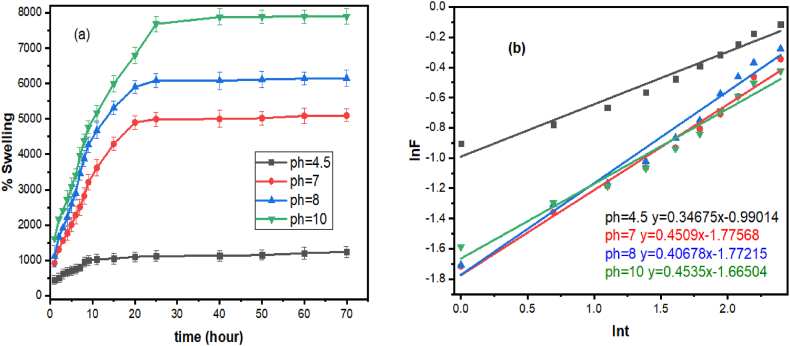
% Swelling (a) ln F verses ln t plots for swelling (b) AMPS(PHE-Ce)/MC-Cu hydrogel.

The AMPS (PHE-Ce)/MC-Cu hydrogels' swelling behavior was examined in a pH series of 4.6–10.2. Initially the hydrogel swelled significantly, peaking at pH 4.6 (1240%), pH 7 (3259%), pH 8 (3749%), pH 10.2 (1339%). The maximum percentage of swelling, however, stayed relatively consistent since the superabsorbent hydrogels absorbed very little extra water as the soaking duration increased. For instance, after reaching to equilibrium, at pH 4.6 the maximum percentage of swelling was approximately 1300%, having a dry hydrogel mass of 0.0321 g and a swelled hydrogel mass of 0.451 g. At pH 7, there was approximately 5098% swelling where mass of dry hydrogel was 0.0321 and mass of swollen hydrogel was 1.667 g, while at pH 8, it reached about 6154% with swollen hydrogel mass of 2.008. At pH 10.2, the maximum swelling percentages was 7898% with swollen hydrogel mass of 2.566 g. This trend indicates that, with continued exposure to a fluid, these hydrogels, as depicted in Fig. 5a, swelling continued till equilibrium. The swelling phenomenon is due to the significant electrostatic repulsion between the –SO_3_H- groups of AMPS [46]. The superabsorbent experiences a substantial increase in swelling at pH 4.6. This is mainly due to the significant expansion of the hydrogel's pores within this pH range. The critical factor here is the formation of carboxylate (–SO_3_H–) anions, which are negatively charged. The pores are forced to enlarge even further by the electrical repulsion produced by these negatively charged groups. As a result, water can pass through the hydrogel without difficulty [47]. The decrease in swelling is due to the attraction of charged groups, leading to the closure of pores, resulting at a lower pH [34].

Fick's law [48] was employed to analyze the permeation of water through the AMPS(PHE-Ce)/MC-Cu catalyst during swelling. The swelling fraction (F) was calculated using Equation (12) based on the swelling data. Subsequently, this swelling fraction (F) was again calculated by Equation (13) to model and gain a more comprehensive understanding of the water diffusion process into the hydrogel.

(12) $$F=\frac{S_{\mathrm{ti}}}{S_{i}}=kt^{n}$$

(13) lnF=lnk+nlnt

Plotting the variables, the constants ‘n' and ‘k' were estimated that characterize the properties of the solvent diffusion and the network of the hybrid material as shown in Fig. 5b. The plots were constructed for comparing the hydrogels' equilibrium swelling (S_i_) and percent swelling at different times (S_ti_). Before the hydrogel reached their maximal swelling, that is, in the first stage when they had absorbed around 59% of the water, this data has been utilized for these plots. The uniform value of ‘n,’ as reported in Table 4 and shown to be 0.41 for hydrogels across all pH ranges, suggests that Fickian type of water diffusion occurs inside the superabsorbent. In other words water diffuses into the hydrogel according to a predictable design that is outlined by Fick's law [48].

**Table 4:** Swelling behavior of AMPS (PHE-Ce)/MC-Cu hydrogels at various pH.

pH	**N**	**K**	χ2 test	**R**^**2**^
4.5	0.351	0.371	0.0075	0.959
7	0.565	0.169	0.0073	0.980
8	0.606	0.169	0.0072	0.978
10	0.495	0.189	0.0075	0.966

#### 3.7.2 Surface morphology

**Fig. 6:**
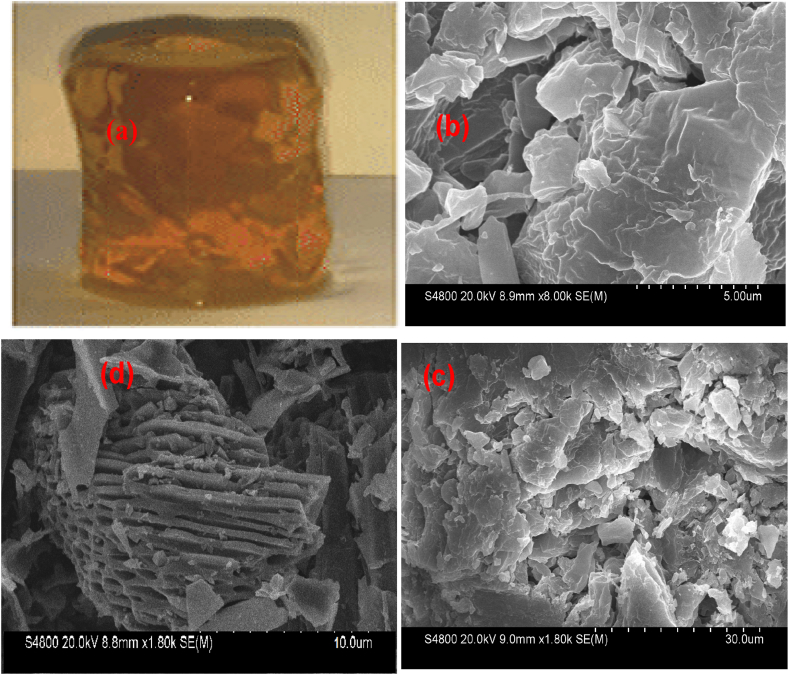
Digital camera image (a) SEM images of surface (b) at 5 μm (c) 30 μm and SEM images of Cross section (d) at 10 μm of AMPS(PHE-Ce)/MC-Cu hydrogel.

Fig. 6a displays images of the hydrogel captured by a camera, while Fig. 6b-d features SEM images of the polymeric hydrogel, AMPS(PHE-Ce)/MC-Cu with copper nanoparticles. Fig. 6b,c, surface image, and Fig. 6d shows a cross-section view of AMPS(PHE-Ce)/MC-Cu. By grafting monomer onto the hydrogel's surface, copper nanoparticles alter its surface. The crosslinking process, which establishes covalent connections between nanoparticles and polymeric chains, imparts a granular texture to the surface of AMPS(PHE-Ce)/MC-Cu, characterized by small pores or fissures [49,50] which improves the hydrogel's ability to draw in and effectively eliminate dyes.

#### 3.7.3 FTIR analysis

**Fig. 7:**
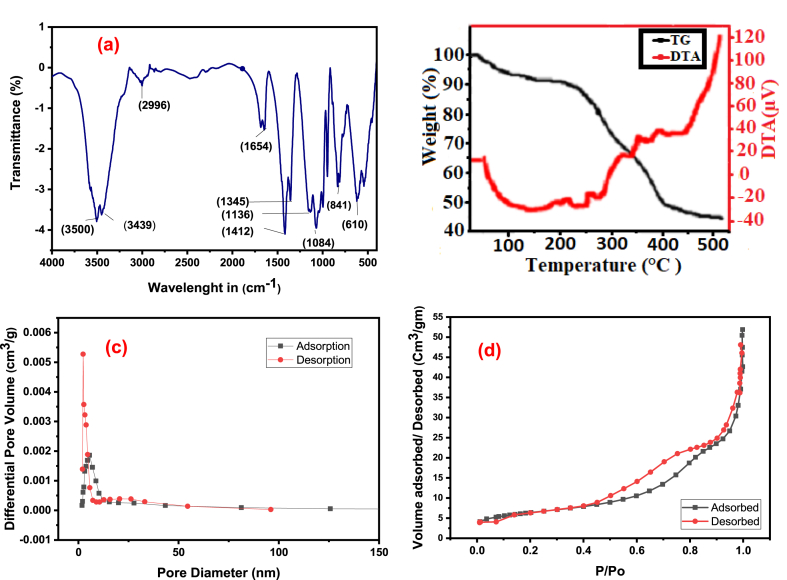
FTIR spectra (a) TGA Curve (b) BJH Pore volume (c) and BET isotherm (d) of AMPS-(PHE-Ce)/MC-Cu.

FTIR spectrum is shown in Fig. 7a. A peak around 2996 cm^−1^, indicating the presence of CH_3_ bonds, which are frequently present in aliphatic molecules. Strong, noticeable peaks were also seen at 3500 and 3439 cm^−1^, which were linked to presence the NH or OH groups in the carboxylic and hydroxyl functional groups of acrylamides stretching. Peaks at 1650 cm^−1^ confirms that carbonyl groups (C=O) present inside carboxyl groups.

Peaks located at 2414 and 1084 cm^−1^ demonstrated the cellulose's -C-O-C groups' bending and stretching vibrations. Moreover, the stretching of C–N bonds was indicated by the peak at 1412 cm^−1^. Additional peaks (1412, 1345, 1084, 1070, 841, and 610 cm^−1^) corresponding to different bending and vibration modes due to the incorporation of Cu nanoparticles. The C–H bonding, vibration, CH_3_ methyl (rocking), C–H (scissoring), C–O stretching, and NH wagging vibration inside the Cu hydrogel were the causes of these extra peaks.

#### 3.7.4 Thermal analysis

The AMPS(PHE-Ce)/MC-Cu Hydrogel was exposed to temperature variations through TGA and DTA tests, as illustrated in Fig. 7b and Table S1. The hydrogel undergoes breakdown into three distinct phases, as indicated by the TGA curve. Firstly, at approximately 100 °C, the first phase involves the loss of about 5.21% of its weight due to the evaporation of both bound and free water molecules [51]. Subsequently, at 203 °C and 330.8 °C, there is a mass reduction of 18.56%. This phase is associated with the breakdown of cellulose present in the gel's structure [52]. Finally, at 466.7 °C, there is a progressive weight reduction of 23.53% until it reaches 466.7 °C. This decline is the consequence of the breakdown of amide and carboxyl groups found in the AMPS component of the AMPS-(PHE-Ce)/MC-Cu Hydrogel [53,54].

Fig. 7b displays the results of the differential thermal analysis (DTA) conducted on the AMPS(PHE-Ce)/MC-Cu Hydrogel. An exothermic peak is notably observed at 425 °C. The thermal degradation recorded in the thermogravimetric (TG) curve within the temperature range of 203 °C–326.3 °C corresponds to the exothermic peak occurring at 302 °C. Another decomposition region in the TG curve correlates with exothermic peaks detected at 433 °C. Furthermore, the DTA curve of the AMPS(PHE-Ce)/MC-Cu Hydrogel exhibits two minor temperature peaks: an endothermic peak at 159.4 °C and another at 255.6 °C [55,56].

#### 3.7.5 BET analysis

One of the popular techniques for figuring out a material's surface area is BET surface area analysis which is used to learn about the size and distribution of its pores. The outcomes of performed analysis are presented in Fig. 7c and d, and Table S2. The process involves studying how the material interacts with nitrogen gas, particularly how it absorbs and releases this gas. When the method was applied to hydrogel, it can be seen that the way it takes in and releases nitrogen falls into two categories called Type I and Type II isotherms. As temperature was raised the area enclosed within the graphs becomes larger. This indicates that the range of pore sizes in the material becomes more diverse.

#### 3.7.6 Energy-dispersive X-ray spectroscopy (EDX)

**Fig. 8:**
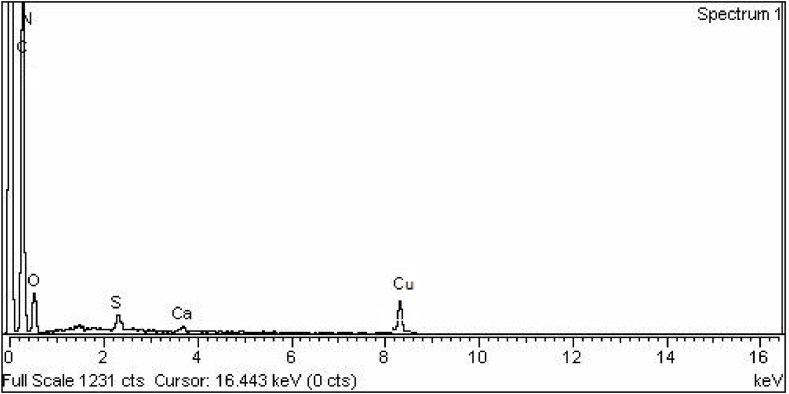
EDX analysis of AMPS(PHE-Ce)/MC-Cu Hydrogel sample.

EDX, also known as Energy Dispersive X-ray Spectroscopy, is a tool employed to determine the elements present in given a sample. EDX analysis revealed peaks corresponding to Carbon, Nitrogen, Oxygen, and Copper in the spectra. The weight percentages of C, N, O, and Cu in the synthesized hydrogel were determined as 57.69, 15.56, 18.21, and 7.21, respectively as shown in Table S3. The EDX spectrum recorded from this sample indicates the presence of signals associated with the elements constituting the copolymer, along with signals confirming the existence of Cu in the hydrogel composition (Fig. 8). Similar results are depicted in the literature as well [57,58].

### 3.8 Reusability and comparison the reported adsorbents

**Fig. 9:**
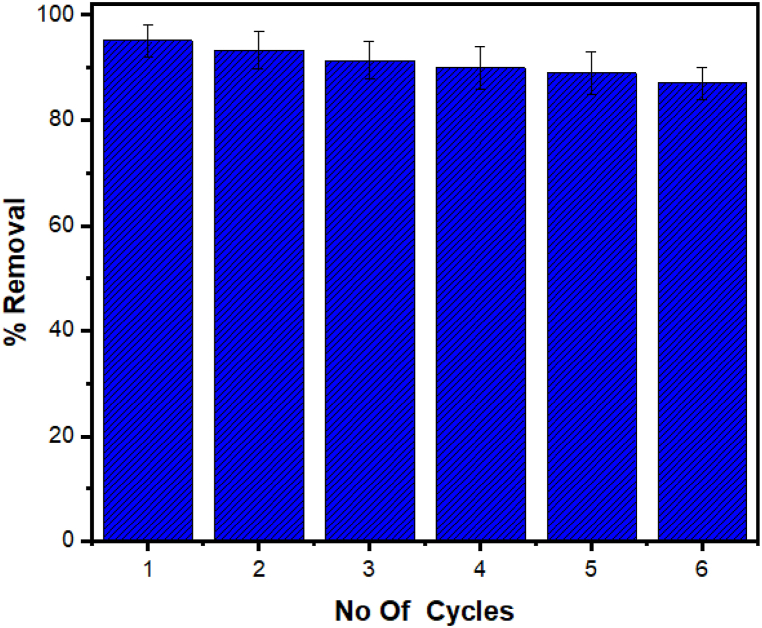
Reusability of copper nanoparticles-based hydrogel.

Fig. 9 highlights the AMPS(PHE-Ce)/MC-Cu hydrogel's recycling capabilities. Following the completion of six sorption-desorption tests, it became evident that these hydrogels still exhibit higher efficiency as sorbent compared to traditional materials. One of their key advantages is their ease of regeneration through a straightforward, one-step desorption process utilizing acetone. In this work, a solvent extraction technique was utilized to successfully renew the BB-3-loaded hybrid sorbent. Approximately 10.7 mL of acetone was used to treat the utilized dye loaded composite material. The entrapped BB-3 molecules were dissolved, allowing the sorbent to be reused. However, it's worth noting that during the recycling process, some MC and Cu nanoparticles were detached. Consequently, compared to the first test, the hybrid material's removal of BB-3 ratio decreased by 18%.

A comparison of the fabricated adsorbent with those reported in literature have been summarized in Table 5.

**Table 5:** Comparison of adsorption capability of AMPS(PHE-Ce)/MC-Cu hydrogel with other adsorbents.

Adsorbent (Hydrogel)	Adsorption capacity (mg.g^−1^)	References
nFeMCH	833	[59]
nFeMCH	1430	[59]
Chitosan	303.21	[60]
(Dex-MA/PAA	1532	[61]
St-g-PAA/APT	1016	[62]
PEE-Gel	1540.19	[63]
Hydrogel based on starch graft acrylic acid	260	[64]
(GG-co-AAc)	1250	[65]
AMPS(PHE-Ce)/MC-Cu	1590	Present work

## 4 Conclusion

An effective and eco-friendly hydrogel, synthesized from acrylamide, cellulose, clay, and copper salt in a specific ratio (69% AMPS, 26% PHE-Ce, and 5% MC) using the free radical polymerization process, abbreviated as AMPS(PHE-Ce)/MC-Cu. The synthesized hydrogel underwent thorough examination through extensive analysis techniques, including scanning electron microscopy, UV/visible spectroscopy, Fourier-transform infrared spectroscopy, thermogravimetric analysis, Energy-Dispersive X-ray Spectroscopy and BET surface area analysis. The SEM pictures clearly revealed that the hydrogel has a rough and granular surface, making it well-suited for absorbing harmful dyes. FTIR spectra showed peaks of various functional groups like OH, C=O, NH, C–O–C, and C–N, etc. required for efficient adsorption to occur. TGA and DTA displayed gradual mass losses in three distinct stages. BET surface area analysis was used for pore size and surface area estimation using BJH and DR methods. The hydrogel has a high water-absorbing capacity of 4536% at about pH 10 with an equilibrium time of 25 h. The optimum pH for adsorption observed was 4.5. Furthermore, Fick's equation was utilized to examine the mechanism of water adsorption and was found to be a good fit because water diffusion was of the Fickian type. The analysis of the molecular structure, conformation, and dynamics of polymers using solid-state NMR is currently unavailable in Pakistan. However, this data will be included in the forthcoming publication. To understand the adsorption mechanism, a comparison was made between the real adsorption (experimental) values and the predicted (calculated) ones, which suggested the pseudo-2nd-order model to fit well the adsorption kinetics data. Langmuir isotherm was the best one, with the highest R^2^ value (0.999) and lower chi square test value (0.0066) while fitting the isothermal data. The sorption was pH-dependent, and the adsorption capacity of the hybrid hydrogel was 1590 mg/g for BB-3 at a high pH of 10.4. Thermodynamic parameters like ΔG°, ΔH°, and ΔS° were studied at 283 K, 293 K, and 303 K, showing that the process is spontaneous with ΔG° values of −4213.45, −4407.34, and −4608.22 J/mol, endothermic with a positive ΔH° value of 1371.32 J mol⁻^1^. Similarly, the entropy was positive with ΔS° value of 19.70 J mol⁻^1^ K⁻^1^. Furthermore, the hydrogel's capacity for reduction was examined through the application of a reputable model called the Langmuir-Hinshelwood model. It was discovered that the hydrogel's ability to function as a good reducing agent was attributed to the presence of Cu inside its network. The discarded hydrogel was regenerated using 10.7 mL of acetone through the solvent extraction method which still had high adsorption capacity after 6 cycles.

## Data availability

No data was used for the research described in the article.

## Funding statement

Researchers supporting Project Number (RSP2024R45) King Saud University, Riyadh, Saudi Arabia.

## CRediT authorship contribution statement

**Sultan Alam:** Project administration. **Imran Badshah:** Methodology, Formal analysis. **Shahid Khan:** Formal analysis, Data curation. **Luqman Ali Shah:** Validation, Supervision. **Muhammad Zahoor:** Writing – review & editing, Writing – original draft, Supervision, Project administration, Funding acquisition, Conceptualization. **Muhammad Naveed Umar:** Funding acquisition, Formal analysis, Data curation. **Riaz Ullah:** Funding acquisition, Formal analysis, Data curation, Conceptualization. **Essam A. Ali:** Funding acquisition, Formal analysis, Data curation, Conceptualization.

## Declaration of competing interest

The authors declare the following financial interests/personal relationships which may be considered as potential competing interests: Muhammad Zahoor reports was provided by University of Malakand. If there are other authors, they declare that they have no known competing financial interests or personal relationships that could have appeared to influence the work reported in this paper.
