# Supplementary material for: Synthesis and characterization of copper nanoparticle-based hydrogel and its applications in catalytic reduction and adsorption of basic blue 3
Source: Heliyon. 2024 Feb 9;10(4):e25836. doi: 10.1016/j.heliyon.2024.e25836 (PMC10875447; doi:10.1016/j.heliyon.2024.e25836)
Supplement: Multimedia component 1 [file mmc1.docx]

**Supplementary file**

**Synthesis and characterization of copper nanoparticles-based hydrogel and its applications for the catalytic reduction and adsorption studies of basic blue 3**

**Sultan Alam^1^, Imran Badshah^1^, Shahid Khan^1^, Luqman Ali Shah^2^,** **Muhammad Zahoor^3, *^, Muhammad Naveed Umar^4^, Riaz Ullah^5^, Essam A. Ali^6^**

1. Department of Chemistry, University of Malakand, Chakdara Dir Lower, 18800, Pakistan; dr.sultanalam@yahoo.com (S.A.), badshahchem17@gmail.com (I.B.), [shahidk955@yahoo.com](mailto:shahidk955@yahoo.com) (S.K.)
2. National Center of Excellence in Physical Chemistry (NCE), University of Peshawar, Pakistan; luqman_alisha@yahoo.com (L. A. S)
3. Department of Biochemistry, University of Malakand, Chakdara Dir Lower, KPK, 18800, Pakistan; [mohammadzahoorus@yahoo.com](mailto:mohammadzahoorus@yahoo.com)
4. Department of Chemistry, University of Liverpool, UK. Email: [m.naveed-umar@liverpool.ac.uk](mailto:m.naveed-umar@liverpool.ac.uk)
5. Department of Pharmacognosy, College of Pharmacy, King Saud University, Riyadh, Saudi Arabia; [rullah@ksu.edu.sa](mailto:rullah@ksu.edu.sa)
6. Department of Pharmaceutical Chemistry, College of Pharmacy King Saud University Riyadh Saudi Arabia: [esali@ksu.edu.sa](mailto:esali@ksu.edu.sa)

^*^ Corresponding author: [mohammadzahoorus@yahoo.com](mailto:mohammadzahoorus@yahoo.com)

Scheme S1: Proposed mechanism for synthesis of AMPS(PHE-Ce)/MC-Cu hydrogel.

Scheme S2: (a) Protonation and deprotonation AMPS (PHE-Ce)/MC-Cu and (b) BB-3

Table S1: Thermograms of AMPS (PHE-Ce) /MC-Cu hydrogels

| **Percentage Weight loss** | | |
| --- | --- | --- |
| 0<Temp ⁰C<100 | 100<Temp ⁰C<230 | 230<Temp ⁰C<500 |
| 5.1 | 18.45 | 23.47 |

Table S2: BET surface area and BJH pore size distribution

| Surface area (m^2^/g) | | Pore size distribution | | | | |
| --- | --- | --- | --- | --- | --- | --- |
|  |  | BJH Methods | | | DR Method | |
| BET | Langmuir | Pore volume (cm^3^.g^-1^) | Pore diameter (A°) | Micro-pore volume (cm^3^.g^-1^) | Average pore width (A°) | Adsorp. Energy (kJ.mol^-1^) |
| 27.87 | 40.32 | 0.089 | 8.68 | 0.014 | 12.67 | 3.94 |

Table S3: Elemental composition of AMPS (PHE-Ce) /MC-Cu Hydrogel hydrogel

| Element | Weight% | Atomic% |
| --- | --- | --- |
| C | 57.69 | 63.51 |
| N | 15.56 | 14.58 |
| O | 18.21 | 16.30 |
| S | 1.13 | 0.46 |
| Cu | 7.21 | 5.02 |
